# Stem Cell Therapy for Treatment of Ocular Disorders

**DOI:** 10.1155/2016/8304879

**Published:** 2016-05-15

**Authors:** Padma Priya Sivan, Sakinah Syed, Pooi-Ling Mok, Akon Higuchi, Kadarkarai Murugan, Abdullah A. Alarfaj, Murugan A. Munusamy, Rukman Awang Hamat, Akihiro Umezawa, Suresh Kumar

**Affiliations:** ^1^Department of Medical Microbiology and Parasitology, Universiti Putra Malaysia (UPM), 43400 Serdang, Selangor, Malaysia; ^2^Department of Basic Medical Science and Department of Surgical Sciences, Ajman University of Science and Technology-Fujairah Campus, Al Fujairah, UAE; ^3^Department of Biomedical Science, Faculty of Medicine and Health Sciences, Universiti Putra Malaysia (UPM), 43400 Serdang, Selangor, Malaysia; ^4^Genetics and Regenerative Medicine Research Centre, Universiti Putra Malaysia (UPM), 43400 Serdang, Selangor, Malaysia; ^5^Department of Chemical and Materials Engineering, National Central University, Jhong-li, Taoyuan 32001, Taiwan; ^6^Department of Botany and Microbiology, King Saud University, Riyadh 11451, Saudi Arabia; ^7^Department of Reproduction, National Research Institute for Child Health and Development, Tokyo 157-8535, Japan; ^8^Division of Entomology, Department of Zoology, School of Life Sciences, Bharathiar University, Coimbatore, Tamil Nadu, India; ^9^Department of Zoology, Thiruvalluvar University, Serkkadu, Vellore 632 115, India

## Abstract

Sustenance of visual function is the ultimate focus of ophthalmologists. Failure of complete recovery of visual function and complications that follow conventional treatments have shifted search to a new form of therapy using stem cells. Stem cell progenitors play a major role in replenishing degenerated cells despite being present in low quantity and quiescence in our body. Unlike other tissues and cells, regeneration of new optic cells responsible for visual function is rarely observed. Understanding the transcription factors and genes responsible for optic cells development will assist scientists in formulating a strategy to activate and direct stem cells renewal and differentiation. We review the processes of human eye development and address the strategies that have been exploited in an effort to regain visual function in the preclinical and clinical state. The update of clinical findings of patients receiving stem cell treatment is also presented.

## 1. Introduction

Blindness or loss of visual function can be caused by failure of the light path to reach the retina or failure of the retina to capture and convert light to an electrochemical signal before transmission to the brain via optic nerve [1]. The major causes contributing to blindness include age-related macular degeneration (ARMD), diabetic retinopathy, cataracts, and glaucoma [2–4], which are genetically linked [5] and associated with multiple risk factors including diet [6], hypertension [7], pregnancy [8], and smoking [9]. The occurrences of these pathologies increase with the age of the patient and are thus widely spread among aging populations. Blindness is an extensive disease that not only affects the quality of life of the patients themselves but may have a negative impact on the socioeconomic status of their immediate families [10, 11].

Current treatments have aimed at protecting vision and preventing visual impairment by early diagnosis using various methods of intervention such as surgery, ionizing radiation, laser, or drug treatments [12–14]. Despite the efficiencies of these treatment modalities, they do not provide a complete solution to stop the progression to blindness.

Many recent findings from preclinical data have supported the notion that stem cells have the capacity to revive degenerated cells or replace cells in many major diseases including ocular disorders [15–18]. Stem cells are present in all tissues in our body and are self-renewable and capable of maintaining a certain level of differentiation in response to injury for tissue repair [19–21]. We mainly aimed this review at both clinicians and academicians, so we presented the localization of stem cell progenitors with eye development in different regions in the eye, the functions of these progenitors, and the current clinical trials and their exploitation of nontissue specific stem cells as alternative sources for regaining lost vision.

## 2. Gene and Protein Regulation during Eye Development

Eye development involves indispensable participation of the neural ectoderm (NE), surface ectoderm (SE), ectomesenchymal/cranial neural crest cell (CNCC), and modicum of mesenchymal tissues [22]. During the fourth week of intrauterine life, the forebrain gives rise to two bulges called optic vesicles that extend like a stalk and a cup to trigger the surface ectoderm on both sides [22]. The retinal pigmented epithelium (RPE) and neural retina (NR) are developed from outer and inner layer of optic cup, while the optic nerve is developed from optic stalk [22]. The cup tip becomes the ciliary body and iris by integrating with the CNCC [23]. The surface ectoderm is repressible for the lens, cornea, and conjunctiva [24]. The sclera, corneal endothelium, corneal stroma, iridial stroma, and iridial muscles are contributed by the CNCC [25]. The neural ectodermal derivatives of eye are permanent cells and lack the self-renewal, as like other nervous tissues. But unlike other surface ectodermal derivatives, the ocular ectodermal derivatives do lack the self-renewal in the eye during aging which collectively results in various degenerative disorders.

**Figure 1:**
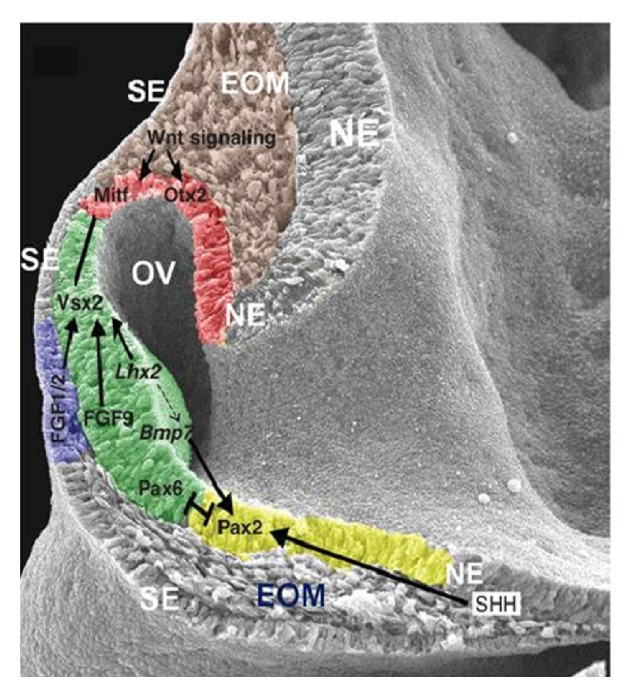
Crucial biomolecules expression in an embryonic mouse at 9.5 days. The neural ectoderm (NE) bulges as optic vesicle (OV) to reach the surface ectoderm (SE) on both sides. The SE became thicker upon the contact of NE to become the lens placode. Except in the lens placode region, the NE and SE are separated by the EOM. In the NE, the presumptive RPE, neural retina, and optic tract are colored red, green, and yellow, respectively. The lens placode is colored blue. The TF reciprocally act to regulate eye development. EOM, extraocular mesenchyme; RPE, retinal pigmented epithelium; NR, neural retina; TF, transcription factor. Copyright 2012. Modified with permission from Cold Spring Harbor Laboratory Press [26].

The well-organized time-dependent interactions and gene expression of all these layers for initiation, pattern determination, and organogenesis are significant for eye development [22, 24–27]. Eye development in an embryonic mouse at 9.5 days is shown in Figure 1 [26]. The neural ectoderm bulges as the optic vesicle to reach the surface ectoderm. The surface ectoderm becomes thicker on contact with the neural ectoderm to become the lens placode. Except in the lens placode region, the neural ectoderm and the surface ectoderm are separated by the extraocular mesenchyme. In the NE, the presumptive RPE, NR, and optic tract are colored red, green, and yellow, respectively, in Figure 1. The lens placode is colored blue in Figure 1. The transcription factors described in Figure 1 are involved in the regulation of eye development.

*Pax6* is a crucial and evolutionarily conserved homeobox gene of eye development [28, 29]. Along with* Pax6*, the other associated genes reported for eye development are* Rx*/*Rax*,* Pax2*,* Lhx2*,* Mitf*,* Otx2*,* Sox2*,* Six3*,* Pitx*,* Vsx2*,* Crx*,* Optx2*, and* FaxL2* [28–39]. The expression of* Pax6* is upregulated by* Six3* and downregulated by Shh (Sonic hedgehog) [35] to help eye formation on both sides [39]. The transcription factor* Pax2* is important for the formation of the optic stalk (which becomes the optic nerve). Retinal axons from both the eyes selectively decussate at the midline named optic chiasma (crucial for vision) which is failed when the* Pax2* mutates (optic chiasma) [23].

Initiation of optic vesicle formation from the neural ectoderm by* Rx*/*Rax* involves extensive cell movements and proliferation [36]. In addition,* Rx* is essential for expression of* Lhx2*,* Pax6*,* Mab2112*, and* Six3*, which specifies the retinal progenitor cells in the optic cup [30, 31, 36, 37].* Lhx2*, a patterning gene expressed in the neural ectoderm, is important for expression of* Mitf* [32].* Mitf* is a governing gene for RPE that specifies the neural retina, and in the neural ectoderm, RPE regulates the vesicle to cup transformation and activates the retinoid acid receptor, which is another important factor for eye development [33, 34]. In* Lhx2* mutant mouse,* Mitf* and* Vsx2* are never initiated and* Pax2*,* Vax2*, and* Rx* are initiated but not maintained, resulting in arrest of eye development in the optic vesicle stage [40]. The optic vesicle is important for lens formation and the lens is important for the vesicle to cup formation. The surface ectoderm will not form the lens if the optic vesicle is removed. In contrast, when provided with an optic vesicle, any primitive ectoderm will develop into the lens [40].

**Figure 2:**
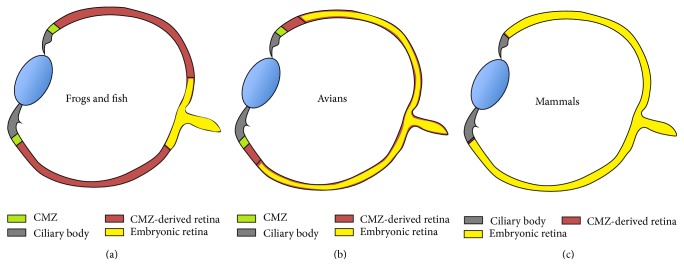
CMZ in vertebrates. The CMZ is progressively reduced in higher vertebrates. The adult eye of different vertebrates (frogs and fish (a), avians (b), and mammals (c)) is shown in blue and represents the neural retina of embryonic origin, which lacks the continuous renewal ability of the CMZ, which is shown in yellow. CMZ: ciliary marginal zone. Copyright 2004. Modified with permission from UBC Press [27].

The neural retina, the brain of eye with nine distinct layers, transmits color signals in and out as vision [22]. During development, the neural retina depends on the expression of* Vsx2*, an important homeobox gene for early patterning and maintenance of cell proliferation and fate [41]. MAPK/FGF signaling is important for neural retina and upregulates* Vsx2* [42] and* Vsx2* downregulates* Mitf* [42–44]. This regulation helps control the distinct neural retina and RPE specification in the optic cup. FGF9, normally expressed in the distal optic vesicle, is important for the boundary between the neural retina and the RPE [44–46]. FGF receptor activation is crucial in chicks but not in mice [46]. This suggests that there is species-specific neurogenesis. Interestingly, specific activation of MAPK/FGF can induce neural retina formation from presumptive RPE with distinct layers [43, 45, 47–49]. BMP is important for Vsx2 regulation [50].  Figure 2 shows a schematic of the adult eye of different vertebrates (frogs and fish, birds, and human) [27]. The ciliary marginal zone (CMZ, yellow color in Figure 2) is progressively reduced in higher vertebrates. Unlike the earlier vertebrates, the neural retina in mammals (blue color in Figure 2) is not renewed continuously because of the absence of the CMZ [51]. The neural progenitor marker* Nestin* is expressed in the junction of the ciliary body with the neural retina, suggesting the remaining of a CMZ even though the relationship is not clear [52]. The regeneration studies reported with RPE to neural retina are akin to transdifferentiation under suitable conditions [53].

Müller glial cells are a progenitor glial component of the neural retina, which arise from activation of* Notch*,* Rax*, and* Jak* signaling pathway [54]. The RPE is an array of uniformly arranged cells in a single layer between the retina and choroid [22]. MITF governs the RPE, the bHLH transcription factor that is the first and critical gene expressed in presumptive RPE and is specific for patterning and cell proliferation [38].* Mitf*, the regulator of the pigmented cells (both in the RPE and in the CNCC) is expressed even before the pigments are formed in the RPE [24].* Mitf* is initially expressed throughout the optic vesicle but is later downregulated in neural retina for layer specification.* Otx2* is important for* Mitf* expression [33].* Pax* regulates both the* Mitf* and* Otx2* [55].* Pax2* and* Pax6* bind and activate the* Mitf* A enhancer [35]. Retinoid acid signaling regulates the optic cup morphogenesis and induces apoptosis in extraocular mesenchyme [33]. Retinoid acid receptors (RAR-*α*,*β*,*γ*) are important for signal transduction of retinoic acid, which is important for the maintenance of the RPE [56]. The enzymes, retinaldehyde dehydrogenases (Raldh) 1, 2, and 3, are vital for retinoid acid synthesis. Raldh 3 originates from the RPE, and Raldh 2 originates from the surrounding mesenchyme [57]. Pitx2 is also important for RPE differentiation [58]. The fate of RPE is influenced by* Shh* [59]. Growth arrest specific 1 (*Gas1*) is a (GPI) protein that binds and coregulates with* Shh* [60].* Gas1* downregulates the proliferation of the RPE to maintain a single cell-layered structure [60]. There are reports of the distinct control mechanisms by BMP in the ventral and dorsal aspects of the RPE [61]. The Wnt/*β*-catenin pathway also controls the optic cup differentiation by activating* Mitf* and* Otx2* [62].

The ciliary body and the iris are developed from the optic cup tip with the incorporated connective tissue stroma derived from the migrated CNCC. The smooth muscles of the iris, namely, the sphincter and dilator pupillae, are derived by transdifferentiation of the pigmented epithelial cells of neural origin [43, 63]. The iris and ciliary body regulate the light reaching the retina and maintain the intraocular pressure by maintaining the aqueous humor secretion [61, 64]. The pigmented cells of the iris possess the ability to differentiate into RPE, neural retina, and lens, and a potential source of stem cells in mammals [65]. FGF, BMP, and Wnt/*β*-catenin participate in the differentiation of progenitor cells into ciliary and iris epithelium [45, 66].

**Figure 3:**
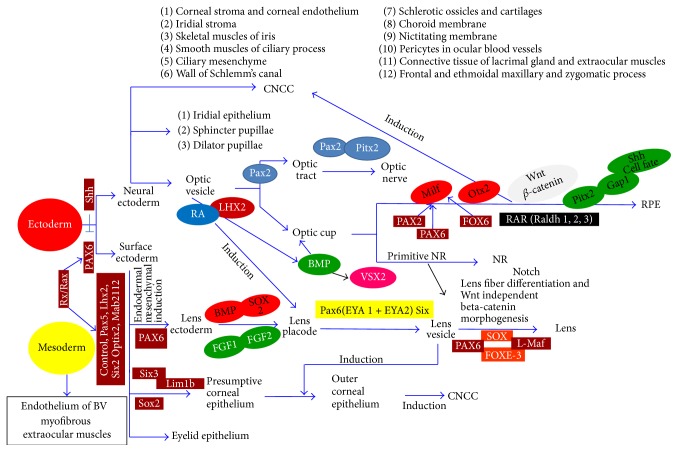
Flow chart: major events of eye development and the involvement of biomolecules. Copyright 2009. Modified with permission from Mosby/Elsevier Ltd. [23].

The lens is derived from the surface ectoderm upon receiving instruction by* Pax6* to respond to FGF, BMP, and* Sox2*.* Fox*-3 helps in the separation of the lens from the surface ectoderm and formation of lens fibers. Lens fibers are epithelial cells that undergo clever modification to become transparent fibers by losing their organelles and accumulating crystalline protein;* Pax6*,* Pitx3*,* c-Maf*,* HSF4*,* RAR*,* Six*,* Sox*, and* Prox* are the transcription factors related to crystalline genes [66, 67]. The CNCC is crucial for eye development and restricts the lens formation area in the surface ectoderm by inhibiting cells other than those for the lens. The lens is under the control of the retina throughout life. The retinal secretion of FGF accumulates in the vitreous humor and stimulates the lens part facing the retina to form lens fibers. If the developing lens is rotated, the cell type changes to form lens fibers from the surface facing the retina (Figure 3) [23]. Attempt can be made to turn the defective lens, front to back to find the results, because the side of the lens which faces the retina is influenced with better survival.

The optic cup is surrounded by mesenchymal cells predominantly of CNCC origin that help in the formation of the vascular coat called the choroid and fibrous coat, namely, the sclera. Transcription factors involved with the scleral development are* Foxc1*,* Foxc2*,* Lmx1b*,* Pax6*,* Pitx2*,* RARb*,* RARg*,* RXRa*,* Six3*, and* Smad2* [30, 31, 36, 39].

The corneal epithelium is continuous with the conjunctiva covering the visible part of the sclera. The junction between the corneal and conjunctiva is named the limbus, which holds stem cells for the renewal of the epithelium throughout life [22]. The corneal epithelium is constantly renewed every 7 to 10 days. Corneal epithelium expresses ΔNp63*α*, ABCG2, integrin *α*9, Bmi-1, EGFR, TGF, and PDGF growth factor indicators for their stemness. The stromal interaction is important for the cell renewal achieved by paracrine factors, hepatocyte growth factor (HGF), and keratinocyte growth factor (KGF). These factors are fibroblast-derived epithelial mitogens of the FGF7 family. In the corneal endothelium the morphology, collagen expression, and cell proliferation are maintained by TGF-*β*1 and TGF-*β*2 [46, 49]. Altogether, the corneal endothelial integrity is preserved by Pax6, Lmx1b, and Pitx2 [37].

The tissues of eye which are commonly associated with diseases are the surface ectoderm derivatives cornea and lens and the neural ectoderm derivatives RPE and retina. Since the lens and cornea do lack the renewal capacity during aging, stem cells from other surface ectoderm derivatives which are relatively easy to collect can be reprogrammed by manipulating target genes and proteins with the help of gained knowledge from molecular biology for regenerative therapy. Regenerated lenses from stem cells can be more exciting personalized regenerative treatment.

The aim of understanding the sequential events during embryogenesis at molecular level cell to cell communication is to understand the pathogenesis and to design the regenerative or genetic therapy to restore normal. The commonest degenerative eye disorders can be tactfully managed by delivering target proteins to prevent and to repair several types of ocular diseases. Stem cells of eye are closely associated with maxillofacial tissues including dental stem cells (a derivative of CNCC) during embryogenesis which retain the stem cells till life can be traced and reprogrammed for stem cell therapy. Researchers differentiated retina [68] from dental pulp stem cells. Epigenetic memory explains that the differentiated cells retain the memory of their original tissue and on reprograming they spontaneously dedifferentiate to its original tissue [69, 70]. If the suggested RPE differentiation from dental stem cells [71] is succeeded, it will be more acceptable than the controversial embryonic stem cells, which has proven its success after 2 years of follow-up of clinical trial [72, 73]. Autologous oral mucosal epithelial cells have been successfully reconstructed to fabricate cornea to restore vision [74].

## 3. Manipulation of Stem Cells for Cell Replacement Therapy for Treatment of Ocular Disorders

To dispense a suitable intervention, the mechanism that regulates cell renewal, differentiation, and maturation change in a diseased microenvironment needs to be understood. One of the major inherited ocular disorders, Retinitis Pigmentosa (RP), is characterized by progressive degeneration of photoreceptors in the retina [75–78]. Complete blindness in most cases proves that humans lack a homeostatic mechanism to replace lost photoreceptors [79].

The earliest interventions used autologous tissue resident stem cells such as RPE cell suspensions or RPE-choroid sheets to improve vision of patients affected by age-related macular degeneration via subretinal translocation [80]. Other sources of stem or progenitors cells from extraocular tissues such as hematopoietic stem cells (HSCs) [81–83], dental pulp stem cells (DPSCs) [68], hair follicle stem cells (HFSCs) [84], mesenchymal stem cells (MSCs) [76, 85–89], and induced pluripotent stem cells (iPSCs) [90–92] have been explored for regenerating retinal neurons, corneal or conjunctival epithelial cells, and the RPE. The reason for using these stem cells is their capability to form neural progenitor cells or mature optic cells and the release of trophic factors important for reparative mechanism (Table 1 [76, 82, 83, 85–88, 90, 91, 93–99]). The manipulation of these cells raises less debate over moral and ethical issues than the use of ESCs [93] and fetal stem cells [100, 101]. Moreover, the eye is a suitable target organ for stem cell transplantation because it is immune-privileged, and strict containment by the blood-retinal barrier will disable the emigration of possibly maltransformed injected cells to extraocular tissues [102].

**Figure 4:**
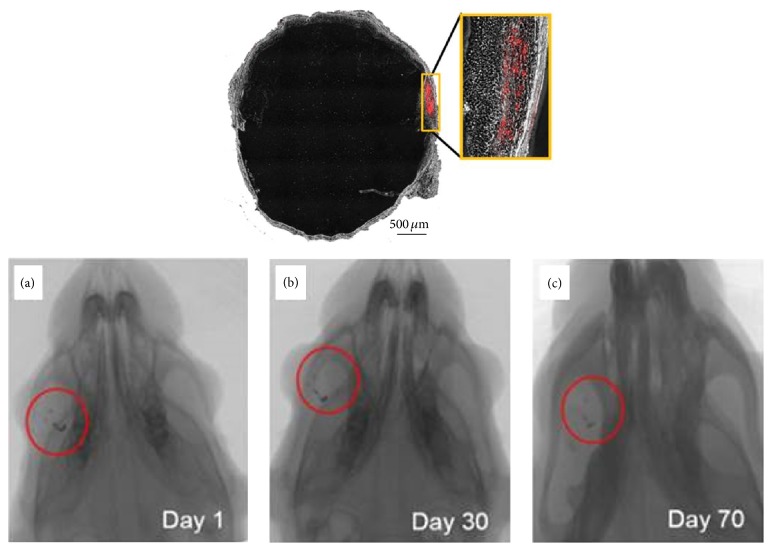
Tracking of injected human Wharton's jelly-derived MSCs in an RP rat model (Royal College of Surgeons rats) with microcomputed tomography. Microcomputed tomography images show localization of gold-loaded human Wharton's jelly-derived MSCs in the right eye (a) on day one. The cells were found to be retained in the eye without further migration at day thirty (b) and day seventy (c) after transplantation. PKH 26 (labelled red) indicated the subretinal site of human Wharton's jelly-derived MSCs after cell transplantation at week two. Modified with permission from Creative Commons Attribution License [103].

**Figure 5:**
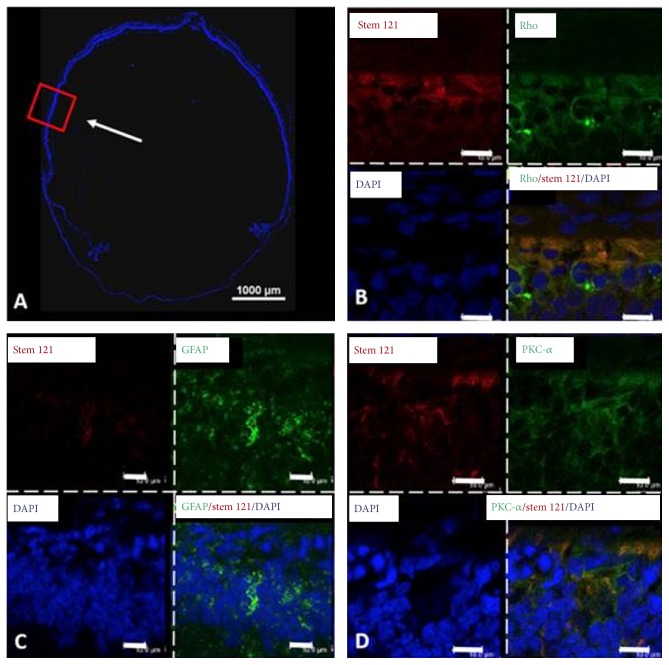
Observation of differentiation of human Wharton's jelly-derived MSCs into retinal cell phenotypes in RCS rats by confocal microscopy. Confocal microscopy picture of the whole eye (A) and magnified pictures of the transplanted region (B–D). The red box indicates the magnified region, and the white arrow demonstrates the transplanted region. The antibodies used were anti-PKC-*α* (bipolar cell), anti-human/rat rhodopsin (rod photoreceptor), anti-human stem 121 (MSC), and anti-GFAP (Müller glial cells). DAPI was used to stain the nucleus in the retinal layer. Colocalization of DAPI (blue) and stem 121 (red) with PKC-*α* (green), GFAP (green), and rhodopsin (green) was found at day seventy after transplantation, suggesting that human Wharton's jelly-derived MSCs have the ability to differentiate into retinal neurons or to fuse with the degenerating neurons. Scale bar indicates 10 *μ*m. Modified with permission from Creative Commons Attribution License [103].

Figure 4 shows microcomputed tomography images to track the injected human Wharton's jelly-derived MSCs (hWJ-MSCs) in a Retinitis Pigmentosa rat model [103]. The gold-loaded hWJ-MSC remained in the eye with no systemic migration to other organs detected on day 70 after injection. This study indicated that the injected MSCs were confined to the subretinal layer of experimented eyes and that no systemic migration occurred in the rat model [103].  Figure 5 shows rat retinal cell phenotypes exhibiting modest level of human MSCs marker, as observed by confocal microscopy. Colocalization of stem 121 (mesenchymal stem cell marker, red color in Figure 5) with rhodopsin (green), GFAP (Müller glial cells, green color in Figure 5), and PKC-*α* (bipolar cells, green color in Figure 5) [103] was found, implying that MSCs could have differentiated into specific retinal cell phenotypes upon activation by cytokines released by the dying cells or fused with the degenerating cells to rescue tissue death [104, 105]. It is noteworthy that other studies have also demonstrated differentiation of human Wharton's jelly-derived MSCs into neurons [106], glia [107], and retinal progenitor cells [108]. Hence, introduction of hWJ-MSCs might be beneficial in inducing certain level of cell repair or regeneration in retinal degeneration.

However, the most significant barrier for successful visual restoration has been the failure of these neuron-derived stem cells to integrate into the retinal circuitry. In central nervous system, stem cells and its neuron derivatives were reported to successfully integrate into the host neural circuitry [109–112]. On the contrary, the integration of transplanted cells might be influenced by the molecular predisposition in the damaged eye tissues, which could vary even between different regions [111] and the ontogenetic stage of transplanted neurons [104]. MacLaren et al. first demonstrated that physiologically older retinal tissues showed predilection and tissue acceptance to later ontogenetic stage of transplanted retinal cells, that is, immature postmitotic photoreceptors over neural progenitor cells [104]. Human ESCs-directed differentiated retinal cells could migrate and integrate into the retinal layer and form synapses in *CRX* ^−/−^ transgenic mice following intravitreal injection at birth or postnatal day 1 [113]. Conversely, there is also a report that ESCs-derived neural stem cells showed lesser migration and integration in the retina. To prove that ontogenetic stage of transplanted neurons would also determine the level of integration, West et al. used three-dimensional culture of mouse ESCs with overexpression of* Rax* genes to direct generation of retinal neuron cells at different time points to establish an equivalent retinal developmental stage for a retinal cell integration study [114]. Unfortunately, their results were not able to prove that the transplantation of photoreceptors at the late ontogenetic stage has better integration into the retinal layer. However, a significant reduction of tumorigenic formation in the retina was observed when photoreceptors were used than when ESCs were used. The difference in the gene expression profile of the different ontogenetic stage of stem cells or progenitors may not mimic the native characteristics of retinal neurons, hence, an incomplete integration into the retinal circuitry. The characteristics of transplanted cells can be significantly affected by the choice of culture methods [115]. Generally, future studies should widen focus on the determination of geographical protein expression in different ocular disorders and identification of similarities in gene expression, rather than mere dependence on morphological observation or* in vitro* functional studies. It is hoped that these efforts would provide clue on tissue predilection over specific stem cells or its neuron derivatives for maximum therapeutic efficacy.

**Figure 6:**
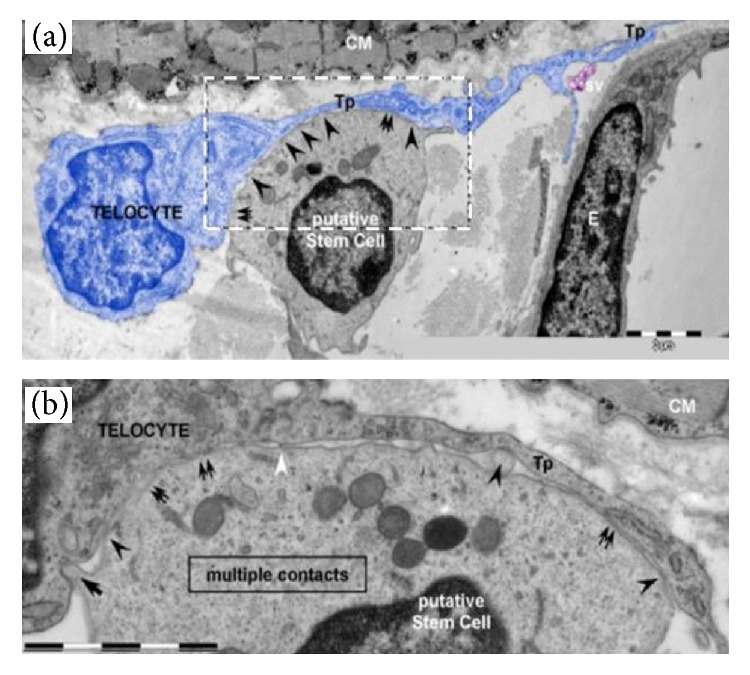
Electron microscopy pictures of telocyte-putative stem cell junctions observed in the human heart. The picture describes the contact point (arrowheads) among a putative stem cell and a telocyte (blue color). Broader planar contacts (double arrows) can be observed. (a) The average distance between the putative stem cell and plasma membranes of telopode, Tp, is 43 ± 20.3 nm (min: 20.3 nm; max: 90.6 nm). E, endothelial cell; sv, shed vesicles; CM, cardiomyocyte. (b) High magnification on a consecutive ultrathin region of the rectangular site indicated in (a) describes the geometry of the 8 *μ*m long heterocellular connections; plasma membranes of tight-fitting apposed sectors (double arrows); dot contacts (arrowheads) change with planar contacts. A small cellular projection of putative stem cell (arrow) is located on a small recess of the telocyte. Thick nanostructures (15–20 nm) can be found with connection of the plasma membranes of the cells (white arrowheads). Bars represent 2 *μ*m. Modified with permission from Creative Commons Attribution License [116].

There is also a suggestion that concomitant transplantation of stem cells with telocytes may help restore the microenvironment. Telocytes are interstitial cells that reside in close contact with stem cells (Figure 6) and may be responsible for the transfer of bioactive molecules (nutrients and paracrine factors) among neighboring cells such as nerve cells and blood vessels [116]. The presence of telocytes has been reported in skeletal muscle [116], uterus [117], skin [118], heart [119], digestive tract [120], lung [121], and iris and uvea of mouse eyes [122].

Advanced techniques have also used a denuded amniotic membrane as a substratum for epithelial cell culture and stratification [123] and used cord blood serum to replace xenobiotic material [124] for conjunctival or corneal transplant. Recently, there is also research effort in developing a new mode of delivery of stem cells through direct application of contact lenses on the ocular surface [125]. Observation of successful stratified epithelization on a corneal wound bed in a rabbit model of limbal stem cell deficiency following application of modified-contact lens (with plasma polymer with high acid functional group) cultured with limbal cells has high clinical indications, suggesting that surgery for corneal transplant may not be needed in the future [125]. Laboratory procedures are getting standardized with simple protocol for culturing limbal cells to adopt with many cell sources [126]. Markers like Keratin 14 is used to map the distribution of precursor cells of cornea and suggested for corneal renewal with stem cells for alternative regenerative therapy [127]. Also to strengthen the universal standard in techniques, good manufacturer practice based on UK facilities on ocular surface reconstruction is suggested for use outside the UK [128].

## 4. Current Use of Stem Cells for Ocular Disorders

### 4.1. Retinal Degeneration

Retinal degeneration is a medical condition that affects the health and welfare of adults and children in the developed world. It represents a group of blinding diseases that include age-related macular disease, glaucoma, optic neuropathies, and retinal vascular complication. Many clinical trials were performed to develop treatments for these diseases. However, it was reported that those approaches were still unable to entirely cure the disease. Interestingly, a stem cell-based treatment shows an extraordinary potential to rectify some of these diseases. In the past few years, studies strongly propose that stem-cell-based therapy has the ability to correct defective function of retina photoreceptors [114, 126], ganglion cells, retinal pigment epithelium (RPE) [129, 130], and optic nerve [131, 132].

*Retinal Pigment Epithelial Cells (RPE) and Age-Related Macular Disease (ARMD)*. The macula enables people to read, process faces, and drive. Degeneration of the RPE leads to malformation at the macular area of the central vision at the initial phase and eventually progressive loss of central vision. This medical condition, known as age-related macular degeneration (ARMD), contributes to the highest cases of blindness in the elderly population globally [92, 133].

ARMD could be present either in wet or in dry forms (wet and dry ARMD) [134]. Wet ARMD manifests as neovascularization, which can be successfully managed with monthly inoculation of antiangiogenic drugs such as Lucentis [135]. Although effective in treating wet ARMD, the monthly injection into the eye causes discomfort and inconvenience to the patient and is expensive [136]. In contrast, dry ARMD presents as drusenoid aggregates under the basal side of the RPE layer at the early phase. These aggregations will lead to geographic atrophy with pronounced loss of the RPE and photoreceptors at later stage. Most of the ARMD cases (80 to 90% patients) occurred due to the dry form as no effective treatments have been found to date.

**Figure 7:**
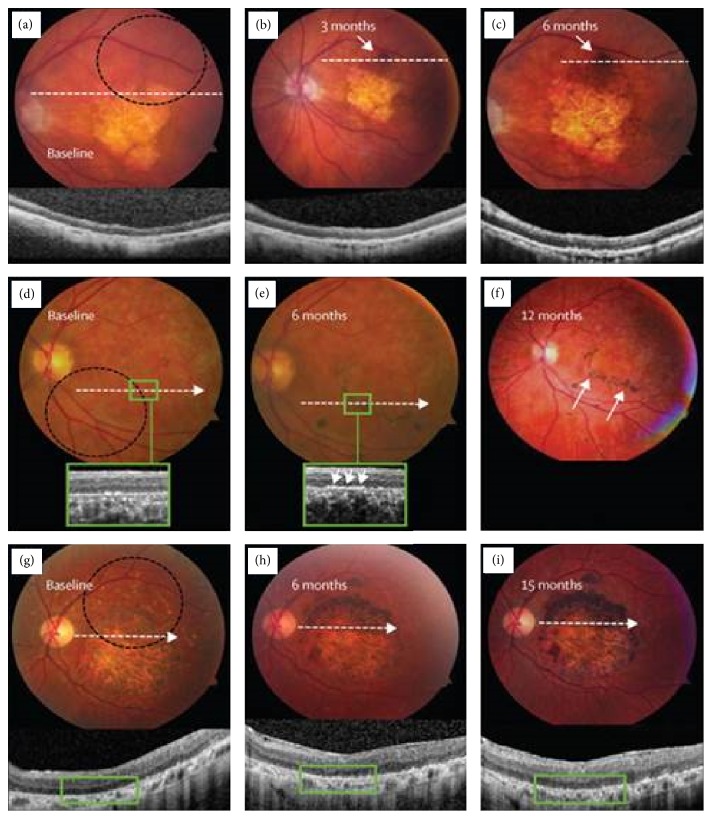
Pictures of eye fundus with pigmentation where retinal pigment epithelium differentiated from human ESCs was transplanted. (a–c) Color fundus pictures and images of spectral domain-optical coherence tomography at baseline of patient eyes of ARMD (dotted circle indicates an outline of the site of cell transplantation) and at an eye after 3 and 6 months of the transplantation. A pigmented patch of transplanted cells (arrows in (b) and (c)) grows bigger and has more pigmentation in six months. Optical coherence tomography (inset of figures) indicates the existence of cells on the inner sites of Bruch's membrane at six months compared with the baseline of the eye. (d–f) Color fundus pictures and pictures of spectral domain-optical coherence tomography at baseline of patient eyes of Stargardt's macular dystrophy (dotted circle indicates an outline of the site of cell transplantation) and an eye after six and twelve months after transplantation. Patches of transplanted cells exist around the edge of baseline atrophy in retinal pigment epithelium (e), which grow more significant after twelve months (arrows in (f)). Pictures of spectral domain-optical coherence tomography at baseline (d) and six months (e) indicate that the enhancement of pigmentation is found at the level of normal monolayer retinal pigment epithelium engraftment, the retinal pigment epithelium, and survival at six months (arrows in (e)), which is close to the site of bare Bruch's membrane being lack of native retinal pigment epithelium. (g–i) Color fundus pictures of a patient of Stargardt's macular dystrophy (dotted circle indicates an outline of the transplantation site). A big central site of atrophy can be seen on the preoperative picture (g). A site of transplantation of retinal pigment epithelium cells can be seen at the superior half of the atrophic lesion at six months (h), which grows bigger and has more pigmentation at fifteen months (i). Copyright 2015. Modified with permission from Elsevier Ltd. [72].

Currently, clinical trials using RPE-derived human from ESCs and other stem cell-derived therapy are ongoing and becoming a promising approach for the treatment of ARMD. Several companies and institutions are actively involved in stem cell research to treat various ocular diseases, including institutions in Japan, USA, Europe, South America, China, Iran, Taiwan, and South Korea. To date, stem cell therapies have been administered to over 200 patients globally. Schwartz and his colleagues [72, 130] performed clinical trials on patients affected by dry ARMD (NCT01344993) and Stargardt's macular dystrophy (NCT01345006) [72]. In these trials, the researchers injected 50,000 to 200,000 hESC-derived retinal pigment epithelial cells into the worst-affected retina of the patients.  Figure 7 shows fundus images taken from the patients following transplantation with hESCs-derived retinal pigment epithelial cells. There were increases in the area size and subretinal pigmentation of patches of transplanted cells in 72% of the treated patients with dry ARMD and Stargardt's macular dystrophy at 3–15 months later [72]. Figures 7(b) and 7(c) showed that the patch of transplanted cells, which were present typically at the boundary of atrophic lesion on the eye of dry ARMD patients, became larger and more pigmented within 6 months. Meanwhile, in a patient with Stargardt's macular dystrophy, patches of pigmented cells were found around the boundary of baseline atrophy in retinal pigment epithelium layer (Figure 7(e)) and appeared more prominent after 12 months of transplantation (Figure 7(f)).  Figure 7(g) shows preoperative image of another Stargardt's macular dystrophic patient with a large central area of atrophy. Six months later after transplantation, the superior half of the atrophic lesion was totally filled in by the transplanted retinal pigment epithelial cells (Figure 7(h)). The filled area became larger in size and more pigmented sites were seen after 15 months of transplantation (Figure 7(i)) [72]. It is important to emphasize that the vision-related quality of life was enhanced in both patients of atrophic ARMD and Stargardt's macular dystrophy. None of the patients have reported signs of abnormal tissue formation at either the local or ectopic site of injections or immune rejections even four months after injection [72].

It should be mentioned that Professor Takahashi's group [137–141] at Kyoto University has been studying the transplantation of retinal pigmented epithelium cells into ARMD patients, which are differentiated from human iPSCs reprogrammed from patient cells. The tissue has maintained its brownish color, which is a sign that it has not been attacked by the immune system [142].

Ocata Therapeutics (formerly known as Advanced Cell Technology) has sponsored the trials at the Jules Stein Eye Institute, Massachusetts Eye & Ear, Wills Eye Institute and Bascom Palmer Eye Institute. Neurotech Pharmaceuticals (NCT00447954) has conducted a trial using encapsulated, modified human RPE cells to express ciliary neurotrophic factor for intraocular implantation into ARMD patients. Another report (NCT01518127) shows that Siqueira has been engaged in wet ARMD treatment using bone marrow-derived stem cells in a prospective phase I/II clinical trial [143]. Unfortunately, the complete outcomes have not yet been posted in the clinical trial registry of US National Institutes of Health (ClinicalTrials.gov) despite the fact that the trial was ended in December 2015. Other institutes, such as CHA Bio & Diostech (NCT01674829), Janssen R&D (NCT01226628), University of California, Davis Eye Center (NCT01736059) [83], University College London, Moorfields Eye Hospital (NCT01691261) [144], Hollywood Eye Institute (NCT02024269), and Stem Cells Inc. (NCT01632527) [145], were also engaged in stem cell therapy for ARMD.

### 4.2. Glaucoma

Glaucoma is the most common neurodegenerative disease in the inner part of retina. Prevalence models predict an increase of glaucoma incidence to 79.6 million by 2020 worldwide, a jump from 60.5 million in 2010 [11]. Similar to other neurodegenerative disorders, the loss of the nerve cell population from the central nervous system can be used to predict the risk of glaucoma. Additionally, signs of glutamate toxicity, oxidative stress, impaired axonal transport, and reactive glial changes are also well-characterized in glaucoma [146, 147]. However, in glaucoma, retinal ganglion cells (RGC) predominantly die, which leads to the degeneration of the optic nerve and disconnecting the communication of signals from the retina to the brain.

Increases in age and raised intraocular pressure can lead to the occurrence of glaucoma. Diagnosis and prescription of a suitable treatment for glaucoma can be too late as patients may present asymptomatically until the end stage of the disease, which results in significant loss of visual function. Clinically verified treatments such as medication and eye surgery could delay the development of the glaucoma by reducing intraocular pressure but fail to halt the disease entirely to prevent loss of vision [148]. As of the date of this review, two registered clinical trials (NCT01920867 and NCT02330978) are recruiting patients for glaucoma treatment with bone marrow-derived mesenchymal stem cells. The safety of autologous stem cells derived from adipose tissue is also currently being tested in a phase I/II clinical trial (NCT02144103) for glaucomatous neurodegeneration. Additionally, Dr. Goldberg at the University of California has tested the treatment of ciliary neurotrophic factor on primary open angle glaucoma patients at the Bascom Palmer Eye Institute, University of Miami (NCT01408472). Several preclinical models have proven that ciliary neurotrophic factor could augment the survival and renewability of retinal ganglion cells [149, 150].

### 4.3. Optic Nerve Disease

The optic nerve can lead to various pathologies due to intraorbital, intracranial, intrinsic, or systemic disorders. Optic nerve diseases could also lead to life- and vision-threatening conditions [151]. Neural loss from the optic nerve is a frequently occurring, irreversible blinding pathology that involves optic light-sensing tissue. Similar to the brain, the eye, which is a part of the central nervous system, will not be able to restore neuron loss after the occurrence of disease [148]. The patterns of optic nerve diseases provide information to the researcher to help understand the fundamental pathological activity and establish a method to enhance advanced detection and treatment strategies [148]. Recently, Dr. Jamadar worked in a clinical trial (NCT01834079) at Chaitanya Hospital, Pune, to evaluate the safety and efficacy of using bone marrow-derived autologous cells for treating optic nerve disease. It is hoped that the primary outcome of reducing degeneration of the optic nerve will also lead to improvement in visual function and decreased intracranial hypertension. Neurotech Pharmaceuticals also used similar RPE cell implants to administer CNTF to patients with optic nerve stroke in a separate phase I clinical trial (NCT01411657).

### 4.4. Other Retinal Diseases

Retinal diseases other than the major ocular diseases discussed above also cause problems. These diseases include retinal detachment and retinal vascular complications. Retinal detachment is a medical condition in which the retina separates from the back of the eye. In a case report by Wilkes et al. [152], one in 10,000 people faces this problem per year. As the detachment period increases, the visual recovery reduces at an exponential rate after macula-off retinal detachment [153]. With modern surgical techniques, such as scleral buckling, pneumatic retinopexy, and pars plana vitrectomy, we can anticipate more than a 90% success rate for anatomical repair [154]. Although these treatments show positive results anatomically, the visual result still remains displeasing due to the enduring functional injury to the macula [155].

Clinical trials for treating retinal detachment began in the 1980s. A report by Brinton states that of 106 cases of eye trauma, 55 eyes (52%) attained final visual acuity of 20/100 after surgery [156]. The researchers also found that patients who engaged in later vitrectomy did not achieve a better final visual outcome than those who engaged in early vitrectomy within 14 days of impairment. In a separate study, Burton found that 53% of patients who experienced macula-off retinal detachments and underwent early surgery reached visual acuity of 20/20 to 20/50 [153]. However, patients with long-standing detachments were not able to reap functional benefits after surgery. A case reported by Suzuki and Hirose in 1997 states that after 3 months of total retinal detachment, vision was recovered in a patient with no light perception (NLP) [157]. After undergoing two surgeries, the patient recovered counting fingers (CF) vision. The scientists hypothesized that some retinal receptors were capable of eluding the failure. Although all of these trials showed a positive result in patient visual function recovery, the treatment is applicable to only early stage impairment and is costly and inconvenient. The use of stem cell-based therapy in retinal detachment cases might be one of the alternative treatments for early or late stage retinal detachment. For instance, fibrovascular scarring in ARMD, DR, ROP, and neovascular glaucoma [158] can be attenuated by introduction of MSCs. The scar tissue could prevent reattachment of retina [159]. MSCs could also neutralize reactive oxygen species in injured eye tissue and secrete various cytokines and growth factors including hepatocyte growth factor (HGF), interleukin 10, and adrenomedullin, which has antifibrotic properties [160].

Some of the commonly arising retinal diseases that lead to vision loss are associated with retinal vascular complications. Of these diseases, diabetic retinopathy, retinal vein occlusion (RVO), diabetic macular oedema (DMO), and proliferative diabetic retinopathy (PDR) are of definite epidemiological significance and lead to blindness. Diabetic retinopathy is the third most dominant source of profound visual function impairment and blindness, followed by RVO [161]. In addition to RVO and PDR, ischemic retinopathies are also familiar diseases involved in vasodegeneration. This situation leads to hypoxia, which provokes the release of cytokines and growth factor in neighboring tissues [162] and then leakage of blood vessels and neovascularization, which has a functionally negative effect on optics.

Intravitreal injections of anti-VEGF antibodies and corticosteroids or laser photocoagulation are the contemporary clinical treatments that help attenuate vascular leakage and macular oedema. However, these treatments cause undesirable side effects and do not resolve the fundamental pathology. The vasodegeneration that occurs in the retina is primarily due to the loss of endothelial cells, smooth muscle cells, and pericytes, finally resulting in vascular blockage and hypoxia [162]. An ongoing clinical trial (NCT02119689), which started since 2011, has aimed to study on the impaired function of endothelial progenitor cells in patients of diabetic retinopathy. Stitt et al. hypothesized that the introduction of vascular stem cells such as endothelial progenitor cells can recondition the retinal nerve diseases by repairing and restoring the damaged vessel [163]. EyeCyte Inc. develops endothelial progenitor cells for use as angiogenic therapy in response to clinical indications specific to retinal nerve diseases, particularly those of ischemic diseases [148, 164, 165]. Additionally, the University of Sao Paulo has sponsored Dr. Ruben's trial using intravitreal injections of bone marrow-derived hematopoietic stem cells (CD34^+^ cells) for treating ischemic and diabetic retinopathies (NCT01518842). A subset of CD34^+^ hematopoietic stem cells, which are proangiogenic, could work in synergy with endothelial cells to repair damaged blood vessels.

## 5. Conclusion

Stem cell-based therapy holds an extraordinary prospective in improving the lives of people who suffer from visual disorders. Research in this area will continue to grow to develop new remedies in treating and preventing the problem of vision loss. Interestingly, stem cell-based therapy is not a one-stop general remedy; however, it carries a promising future in producing new biological elements used to treat vision loss.

## Figures and Tables

**Table 1:** Manipulation of different type of extraocular stem cells for treating ocular disorders in preclinical and clinical trials.

Stem cells	Experimental design/research or disease model	Route of injection	Research outcomes	References/sources
Hematopoietic stem cells (HSCs)	Chemically damaged retinal neuron in mice	Intravenous injection	Fusion with ganglion, amacrine, and Müller glial cells, heterokaryons reprogramming, and dedifferentiation into neuroectodermal lineage	Sanges et al. [82]
	Delivery of granulocyte-colony stimulating factor in rats with retina ischemia	Intravenous injection	Apoptosis of retinal cells was reduced and improved visual function Localization of HSCs in the retinal layer	Lin et al. [94]
	Transplantation of human HSCs in mice with acute retinal ischemia-reperfusion injury	Intravenous injection	HSC-treated group of mice showed improved retinal histopathology. However there was no significant difference compared to control mice. No intraocular tumor and no abnormal proliferation of human cells in major organs	Park et al. [83]
	Transplantation in retinal degenerative conditions (atrophic ARMD, Retinitis Pigmentosa) or retinal vascular disease (diabetes, vein occlusion)	Intravitreal injection	Clinical trial to measure primary outcome on adverse events is still ongoing	NCT01736059 (ClinicalTrials.gov)

Induced pluripotent stem cells (iPSCs)	Injection of mouse fibroblast iPSC-conditioned medium	Intravenous injection	Maintenance of retina integrity and function by reducing apoptosis of retinal neurons following photodamage	Chang et al. [91]
	Swine iPSCs-derived photoreceptors	Subretinal injection	Integration of photoreceptors was observed in chemically damaged retina	Zhou et al. [90]
	Generation of 3-dimensional neural retina sheet derived from mouse iPSCs and ESCs for subretinal transplantation into retinal degenerative mice	Subretinal injection	Development into outer nuclear layer (ONL) with completely structured inner and outer segments of photoreceptor	Assawachananont et al. [95]
	Generation of photoreceptor cell from adult mouse dermal fibroblast-derived iPSCs for subretinal transplantation into retinal degenerative mice	Subretinal injection	Development of functional photoreceptor in mice	Tucker et al. [96]
	Generation of RPE sheets from human iPSCs for transplantation into wet ARMD patients	Submacular injection	Pilot safety study involving six patients is currently ongoing. RPE were observed to be retained in patients	Kamao et al. [141]

Embryonic stem cells (ESCs)	*In vitro* differentiation of rostral neural progenitors into retinal neuron cells	Not available	Increased cell expression of *CRX*, S-opsin, and Rho/Rcvrn in hypoxic culture condition, indicating differentiation	Garita-Hernández et al. [93]
	Treatment of patients affected by Stargardt's macular dystrophy and atrophic ARMD with human ESCs-derived RPE suspension	Submacular injection	Improved visual function. No signs of hyperproliferation, tumorigenicity, ectopic tissue formation, and immune rejection were observed	NCT01344993, NCT01345006 (ClinicalTrials.gov)
	Treatment of patients affected by wet ARMD with human ESCs-derived RPE sheets	Intraocular injection	Clinical trial is still ongoing. This method of delivery is hoped to overcome the disadvantages of using ESC-derived RPE suspension	NCT01691261 (ClinicalTrials.gov)

Mesenchymal stem cells (MSCs)	Injection of bone marrow-derived MSCs into a laser-induced ocular hypertensive glaucoma of rat model	Intravitreal injection	Increase in retina ganglion cell (RGC) axon survival and significant decrease in the rate of RGC axon loss normalized to cumulative intraocular pressure exposure	Johnson et al. [97]
	Transplantation of bone marrow-derived MSCs into Retinopathy of Prematurity (ROP) rat model	Not available	Reduced apoptosis in retinal cells with higher expression of neurotrophin-3 and CNTF in ROP rats	Zhao et al. [86]
	Direct topical application of MSCs or MSCs-conditioned medium on cornea for two hours	Corneal surface	Reduced inflammation, opacity, and neovascularization in chemically burned cornea	Oh et al. [85]
	Transplantation of bone marrow-derived MSCs in rats following optic nerve crush	Intravitreal injection	Rescued degeneration of retinal ganglion cells and axon regeneration	Mesentier-Louro et al. [88]
	Transplantation of bone marrow-derived MSCs in alkali-induced oxidative stress rabbit corneas	Corneal surface	Reduced apoptosis in corneal epithelial cells, vascularization, and infiltration of macrophages	Cejkova et al. [87]
	*In vitro* differentiation of adult human bone marrow stem cells with retinal pigmented epithelium cells	Coculture experiment	Differentiated cells expressed neuronal and photoreceptor phenotypes	Chiou et al. [98]
	Transplantation of MSCs overexpressing pigment epithelium derived factor in animal models of choroid neovascularization	Not available	Inhibition of neovascularization and MSCs adopted RPE phenotypes	Liu et al. [76]
	Delivery of human adipose-derived MSCs to light-induced *in vitro* and *in vivo* models	Intravitreal injection	Inhibition of photoreceptor degeneration and retinal dysfunction	Sugitani et al. [99]
	Transplantation of human umbilical cord blood-derived MSCs to neurodegenerative rat model	Intraperitoneal injection	Promotion of regeneration and protection of damaged retinal ganglion cells	Zwart et al. [166]
	*In vivo* delivery of human umbilical cord-derived MSCs to early retinal degenerative rat model	Subretinal injection	Preservation and rescue of photoreceptor degeneration and improvement in visual functions	Lund et al. [167]
	Delivery of ADSCs into atrophic ARMD patients. The cells are harvested from liposuction tissues	Intravitreal injection	Clinical trial to measure primary outcomes on adverse events; visual acuity and visual field analysis is still ongoing	NCT02024269 (ClinicalTrials.gov)

Adipose-derived stem cells (ADSCs)	Injection of BMSCs in patients with advanced ARMD (atrophic or neovascular)	Intravitreal injection	Clinical trial to measure primary outcome on visual acuity is still ongoing	NCT01518127 (ClinicalTrials.gov)

Bone marrow stem cells (BMSCs)	Unilateral ocular transplantation into patients with advanced atrophic AMD	Subretinal injection	Clinical trial to measure primary outcome on adverse events is still ongoing	NCT01632527 (ClinicalTrials.gov)

Central nervous system stem cells (hCNS-SCs)	Unilateral ocular transplantation into patients with advanced atrophic AMD	Subretinal injection	Clinical trial to measure primary outcome on adverse events is still ongoing	NCT01632527 (ClinicalTrials.gov)
